# Supplementary material for: DMRforPairs: identifying Differentially Methylated Regions between unique samples using array based methylation profiles
Source: BMC Bioinformatics. 2014 May 15;15:141. doi: 10.1186/1471-2105-15-141 (PMC4046028; doi:10.1186/1471-2105-15-141)
Supplement: Additional file 2 — DMRforPairs output for the comparison of A431-MCF7 and NA17018-NA17105. Please start from the HTML files in each folder. Available via the BMC Bioinformatics website. [file 1471-2105-15-141-S2.zip › 1394847754114233_MOESM2_ESM/A431_MCF7/figures/33996.pdf]

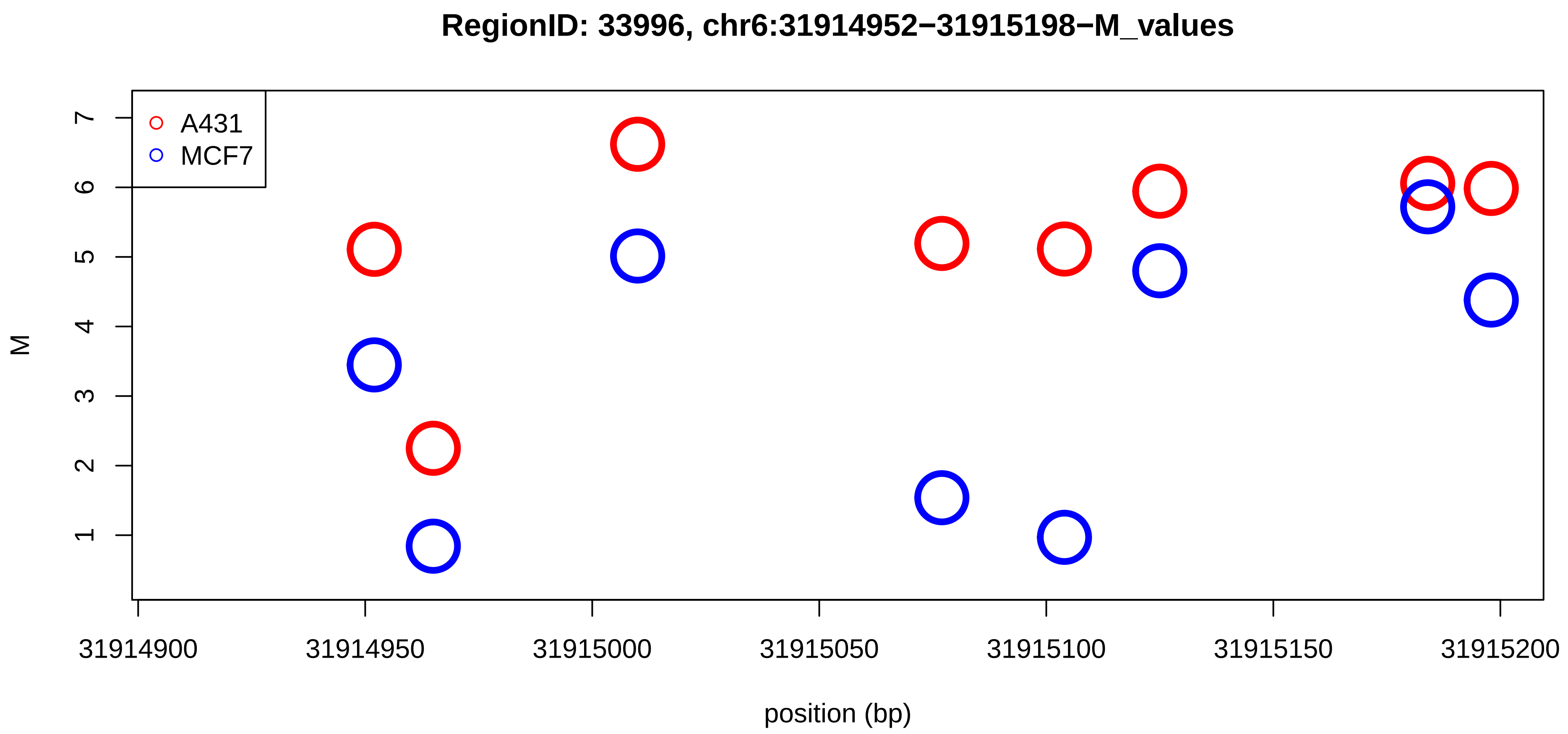

RegionID: 33996, chr6:31914952−31915198−M_values
A431
MCF7
M
1
2
3
4
5
6
7
31914900
31914950
31915000
31915050
31915100
31915150
31915200
position (bp)

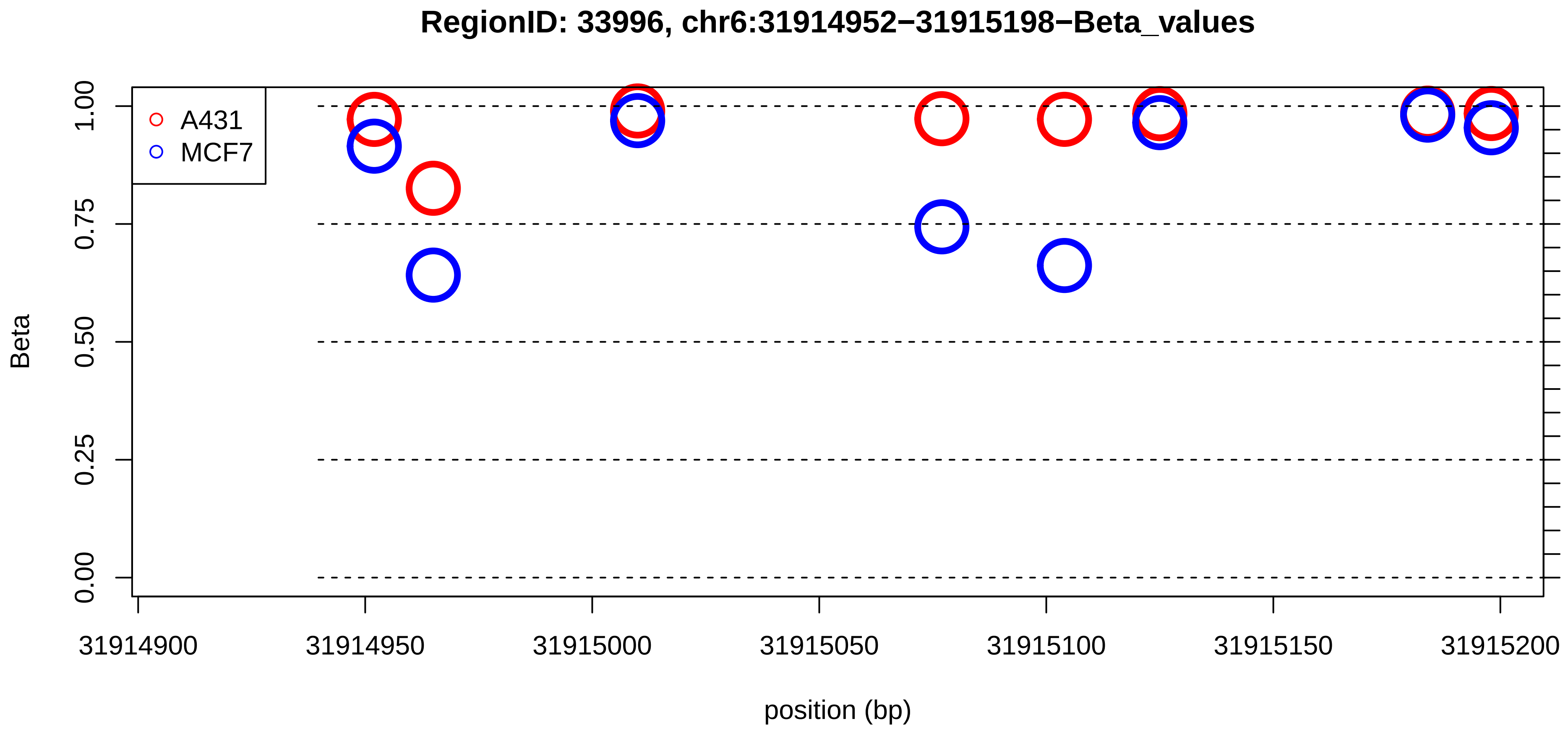

RegionID: 33996, chr6:31914952−31915198−Beta_values
A431
MCF7
Beta
0.00
0.25
0.50
0.75
1.00
31914900
31914950
31915000
31915050
31915100
31915150
31915200
position (bp)

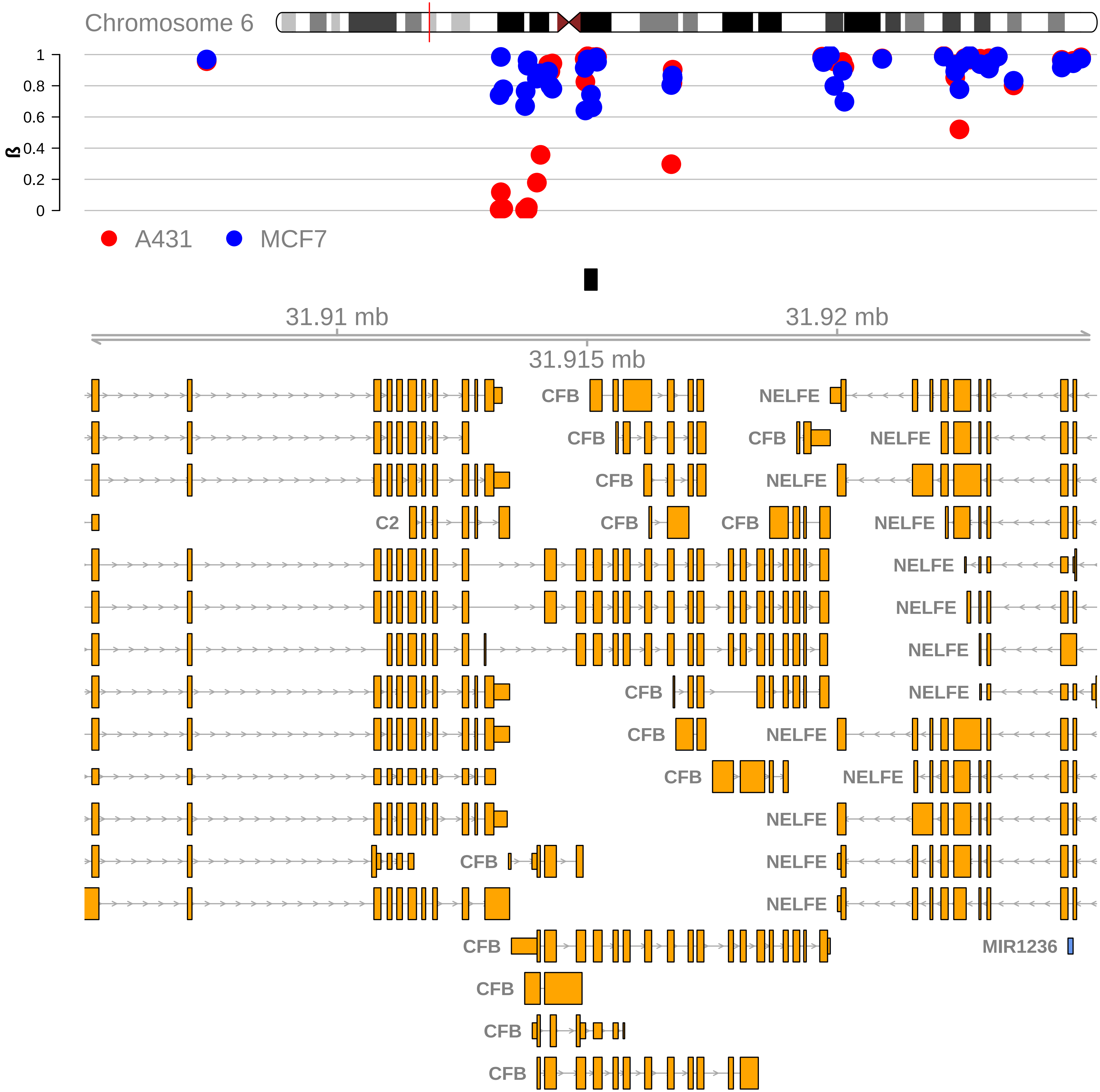

Chromosome 6
ß
1
0.8
0.6
0.4
0.2
0
A431
MCF7
31.91 mb
31.92 mb
31.915 mb
CFB
NELFE
C2
MIR1236
